# Supportive Text Messages to Reduce Mood Symptoms and Problem Drinking in Patients With Primary Depression or Alcohol Use Disorder: Protocol for an Implementation Research Study

**DOI:** 10.2196/resprot.4371

**Published:** 2015-05-15

**Authors:** Vincent Israel Opoku Agyapong, Kelly Mrklas, Victoria Yung Mei Suen, Marianne Sarah Rose, Megan Jahn, Irene Gladue, Jody Kozak, Maureen Leslie, Serdar Dursun, Arto Ohinmaa, Andrew Greenshaw

**Affiliations:** ^1^ Faculty of Health Sciences Department of Psychiatry University of Alberta Edmonton, AB Canada; ^2^ Northern Lights Regional Health Centre Department of Psychiatry Alberta Health Services Fort McMurray, AB Canada; ^3^ Research Priorities and Implementation Research Innovation and Analytics Alberta Health Services Calgary, AB Canada; ^4^ Northern Lights Regional Health Centre Department of Psychiatry Alberta Health Services Fort Mc Murray, AB Canada; ^5^ Nothern Addiction Treatment Centre Department of Addictions Alberta Health Services Grande Prairie, AB Canada; ^6^ Institute of Health Economics and School of Public Health University of Alberta Edmonton, AB Canada

**Keywords:** depression, alcohol use disorder, supportive text messages, intervention

## Abstract

**Background:**

Depression and Alcohol Use Disorders (AUDs) are two leading causes of disability worldwide and are associated with significant treatment challenges requiring new, innovative, cost-effective and technologically-based therapies including the use of supportive text messages.

**Objective:**

To determine the feasibility and effectiveness of supportive text messages in long-term follow-up to reduce mood symptoms and problem drinking in patients with Depression or AUD respectively and to explore the usefulness of self-reports of health services utilization as an outcomes measure.

**Methods:**

This will be a longitudinal, prospective, parallel-design, two-arm, placebo-controlled single-rater-blinded randomized clinical trial with a recruitment period of 6 months and an observation period of 12 months for each participant, with two strata based on primary diagnosis of Major Depressive Disorder or AUD. The sample size will be 120, with about 60 patients randomized from each primary diagnostic grouping. Patients in all intervention groups will receive twice-daily supportive SMS text messages for 3 months and then daily supportive text messages for the next three months. Patients will also receive a phone call every two weeks from the research assistant assigning treatment allocation to confirm that they are still receiving the text messages and to thank them for taking part in the study. Patients in the control group will receive no text messages but will also receive a phone call from the same research assistant every two weeks to thank them for taking part in the study.

**Results:**

The study starts in April 2015 and ends in September 2016. It is envisaged that both qualitative and quantitative primary and secondary outcomes, including patient perceptions of the intervention, will shed light on the feasibility of using automated supportive text message interventions in long term for patients with Depression and AUD. This will inform a full-scale clinical trial.

**Conclusions:**

The paradigm for behavior change using text messages as a patient-direct intervention is consistent with a cognitive behavior therapy approach and addictions counselling principles. Given the automaticity of the messages, we anticipate that if the intervention proves successful, it will represent a low cost strategy that will be readily available and can bring relief to patients in hard-to-reach areas with limited access to psychological therapies.

**Trial Registration:**

ClinicalTrials.gov: NCT02327858; https://clinicaltrials.gov/ct2/show/NCT02327858 (Archived by WebCite at https://clinicaltrials.gov/ct2/show/NCT02327858).

## Introduction

### Background

Depression and Alcohol Use Disorders (AUDs) are leading causes of disability worldwide, associated with significant treatment challenges especially when they occur concurrently [1, 2]. Concurrent challenges include: higher rates of lifetime drug dependence [3]; worse outcomes among those entering treatment for alcohol and drug misuse [4]; higher relapse following AUD treatment among adolescents [5] and adults [6]; greater severity of suicidality in adults [7], and higher likelihood of suicide attempts [8-10] and completed suicides [11]. Given this acute disease burden, we propose a treatment strategy that takes advantage of mobile phone technology to support patients in an efficient, innovative and cost-effective manner. The ultimate aim is to restore health while reducing the combined impact of these disorders on patients, caregivers, their communities and Alberta’s provincial health care provider, Alberta Health Services (AHS) [12].

In a pilot trial in Ireland, participants with concurrent depression and AUD who completed an inpatient dual diagnosis treatment program were randomized to receive twice-daily supportive text messages or a biweekly “thank you” text message for three months. Supportive text messaging improved Beck Depression Inventory (BDI) scores compared with those who received standard care. There was also a trend towards increased Cumulative Abstinence Duration (CAD) in the supportive text message group [13]. Furthermore, the majority of patients who received the supportive text messages expressed satisfaction with the support offered through the technology [14]. The pilot trial in Ireland did not explore the effects of supportive text messages on the individual disorders when they occur alone, although it would be reasonable to assume that they would benefit equally. A recent study in JAMA Psychiatry [15] randomly assigned patients leaving residential treatment for AUDs to either use of a smartphone application to support recovery in addition to standard treatment or treatment as usual. For the 8 months of the intervention and 4 months of follow-up, patients in the smartphone application support group reported significantly fewer risky drinking days than did patients in the control group. In a review of efficacy of use of text messaging as a clinical and healthy behavior intervention, Wei et al (2011) found that among 16 randomized controlled trials, 10 reported significant improvement with interventions and 6 reported differences suggesting positive trends [16]. More recently, in a meta-analysis of the results of text message interventions among 14 studies, the pooled effect size was 0.25, 95% CI (0.13-0.38), indicating that in general, text interventions have a positive effect on reducing substance use behaviors [17].

### Overall Aims

To conduct a randomized controlled pilot trial to determine the feasibility, acceptability and effectiveness of supportive text messages in long-term care to reduce mood symptoms and problem drinking in patients with Depression or AUD respectively.

### Specific Objectives

1. To compare mean changes in BDI scores from baseline for patients with Depression receiving standard care with supportive text messages to those receiving standard care without supportive text messages.

2. To compare the CAD from baseline for patients with AUD receiving standard care with supportive text messages to those receiving standard care without supportive text messages.

### Hypotheses

Supportive text messages will:

1. Lead to a 30% better mean reduction in BDI scores at 6 months from baseline in the intervention group compared to the control group.

2. Increase the mean Cumulative Abstinence Duration (CAD) for patients with AUD in the intervention group from baseline by at least 30% over and above the mean CAD for patients in the control group from baseline.

## Methods

### Study Design and Setting

This will be a longitudinal, prospective, parallel-design, two arm, placebo-controlled single-rater-blinded randomized clinical trial with a recruitment period of 6 months and an observation period of 12 months for each participant with two strata based on primary diagnosis of Major Depressive Disorder or AUD. Patients with AUD will be recruited from those completing the Northern Addiction Residential Treatment program located in Grande Prairie while patients with Depression will be recruited from those assessed for wait listing onto the Cognitive Behavior Therapy (CBT) program run by the Adult Mental Health Team in Fort McMurray. The study has received ethical clearance from the Health Ethics Research Board of the University of Alberta and operational approval from the AHS. All participants will provide informed written consent prior to randomization. The study has been registered with clinicaltrials.gov (NCT02327858) and will be executed according to the timelines specified in the Gantt chart in Table 1.

**Table 1:** Gantt chart for supportive text message project.

		Year 1				Year 2				Year 3			
		Start Date-End Date				Start Date-End Date				Start Date-End Date			
Milestones		Q1	Q2	Q3	Q4	Q1	Q2	Q3	Q4	Q1	Q2	Q3	Q4
**Milestone 1: Recruiting and training of research associates**													
	1.1 Advertising and assembling of research associates	X											
	1.2 Training of research associates and the trainee in psychiatry	X											
	1.3 Writing up a bank supportive text messages in collaboration with service users and CBT and addiction counselors	X											
**Milestone 2: The recruitment of study participants**													
	2.1 Recruitment, baseline assessment, randomization		X	X									
	2.2 Assignment into intervention or control groups		X	X									
	2.3 Delivery of supportive text messages to participants in the control group		X	X	X	X							
**Milestone 3: Follow-up assessment of study participants and collection of administrative data**													
	3.1 Follow-up assessments of individual study participants (Excluding survey on the usefulness of the supportive text messages)			X	X	X	X	X					
	3.2 Follow-up survey of participants in the intervention group on the usefulness of supportive text messages				X	X							
	3.3 Collection of administrative data related to cost of health services utilization						X	X					
**Milestone 4: Data compilation, analysis and preparation of reports, publications and presentations**													
	4.1 Data compilation		X	X	X	X	X	X					
	4.2 Data Analysis		X	X	X	X	X	X	X				
	4.3 Preparation of reports, publications and presentations								X	X			

### Recruitment

All patients completing the Northern Addiction Residential Treatment program located in Grande Prairie and those assessed for waitlisting onto the Cognitive Behavior Therapy program run by the Adult Mental Health Team in Fort McMurray who fulfill the inclusion criteria below will be invited to participate. This will minimize source of referral as a confounding factor. Information leaflets will be provided and those consenting will be recruited. The Structured Clinical Interview for DSM-5 Disorders (SCID) will be used to screen and confirm the diagnosis of all study participants prior to inclusion in the study.

### Inclusion Criteria

Persons aged 18 years and over who are able to provide informed consent will be eligible if they have been assessed and waitlisted for the CBT program in Fort McMurray and fulfill the DSM-5 diagnostic criteria for Major Depressive Disorder or who have completed the residential addiction treatment program located at the Northern Addiction Treatment Centre, fulfill the DSM-5 diagnostic criteria for AUD and are in the stage of change and are committed and motivated to give up alcohol. Participants must be familiar with or willing to learn how to receive text messages and be available for long-term intervention. Mobile phone handsets and top-up call credit of up to 10 dollars monthly will be provided to those who satisfy the inclusion criteria but do not have mobile phones*.*

### Exclusion Criteria

Patients will be ineligible if they do not meet the above inclusion criteria, if they are patients with Schizophrenia, Schizoaffective Disorder, Bipolar Disorder and other psychotic disorders, or residing outside of regular cell phone connection areas.

### Baseline Assessment

All study participants will undergo baseline assessments during recruitment to collect demographic and clinical data. For those with AUD, the time-line follow back (TLFB) [18] will be used to record the quantity of alcohol consumed and number of drinking days during the 90 days preceding the admission to the recovery centre. We will also record any history of alcohol-related pathology. For those with Depression, the BDI will be used to record baseline depression symptom scores. Other clinical measures will include the Obsessive Compulsive Drinking Scale (OCDS)[19] and the Alcohol Abstinence Self-Efficacy Scale (AASES) [20] that evaluates both temptation to drink and confidence to abstain across 20 items representing cues in the four subscale areas of Negative Affect, Social, Physical, and Withdrawal/Urges.

Functional outcome will be assessed by measuring the Health Related Quality of Life (HRQOL). The HRQOL will be measured by the Canadian version of the EQ-5D-5L instrument from all patients. The EQ-5D-5L consists of two components: a five dimensional descriptive system including mobility, self-care, usual activities, pain or discomfort and anxiety or depression with five levels in each of them, and a Visual Analogy Scale (VAS) where respondents value ‘their own health today’ on a scale from 0 (worst imaginable health state) to 100 (best imaginable health state). It is cognitively simple, taking only a few minutes to complete and it can also be used to calculate single index values for economic and population health studies.

### Randomization and Blinding

Randomization will be stratified by primary diagnoses (Depression or AUD) using permuted blocks to ensure balance (1:1) between treatment and control groups within each condition under study. The randomization codes will be transmitted by an independent statistician via text message directly to the researcher. This will commence immediately after the participant has signed the consent form. A dedicated, password-protected phone line with a secure online backup will be used to transmit these messages.

The research assistant who sends the text messages will not be involved in the outcome assessments except to contact those receiving the supportive text messages for feedback on the benefits of the text messages. Participants cannot be blinded and treatment allocation will be made explicit to them. Outcome assessors will be blinded to treatment group allocation. To ensure this, treatment allocation will be concealed from the outcome assessor by blocking access to the database, which contains the randomization code, and all participants in the study will be asked not to reveal their treatment allocation to their assessor. Outside the assessments, outcome assessors will not participate in discussions with the participants in study forums. To test the success of blinding we will ask the assessor to guess the allocation group for each participant at 3, 6, 9 and 12-month follow-ups. Moreover, these assessors will not be involved in data analysis. After data collection is complete all data will undergo a blind review for the purposes of finalizing the planned analysis. Every effort will be taken to avoid incomplete data and methods of dealing with unanticipated missing data will be determined during the blind review of the data.

### Intervention

Patients in all intervention groups will receive twice-daily supportive SMS text messages for 3 months and then daily supportive text messages for the next three months. The messages will be tailored towards improving mood, compliance with medication or targeting abstinence from alcohol in accordance with the primary aims of our study. The majority of the text messages will reflect lessons patients usually learn during CBT and addiction counseling sessions in the two recruitment sites and sent at specified times by a centralized computer program which will be set up and monitored by a research assistant. Sample text messages are outlined below.

- If you keep on going, maintaining your hope and belief that something good will happen, it generally does. One day at a time.
- Ask for help. You have lent a hand to others in need in the past. There is probably someone in your life that would be genuinely honored to help you now.
- Give yourself the time you need to recover, do not try to race ahead to where you think you should be, remember that time is a great healer.
- Think of your recovery as an opportunity to find new solutions in your life. Remember that the past is gone and what you do next is what really matters.

To safeguard patient confidentiality no identifiable patient information will be transmitted via text messages. Patients will also receive a phone call every two weeks from the research assistant assigning treatment allocation to confirm that they are still receiving the text messages and to thank them for taking part in the study. Patients in the control group will receive no text messages but will also receive a phone call from the same research assistant every two weeks to thank them for taking part in the study. The aim of this will be to help increase the retention rate for the study.

### Sample Size

Consistent with the idea that this is a pilot study, the research will use the data that can be elicited from participants who can be enrolled within the existing resources [21]. The study will therefore be limited to a sample size of 120, with about 60 patients randomized from each primary diagnostic grouping.

### Follow-up Assessments

At 3, 6, 9 and 12 months, a blinded researcher (a research psychologist) will contact all patients over the phone and assist them in completing a range of assessment tools relating to the primary and secondary outcome measures. The research assistant who randomized the patients will contact patients in the intervention group by telephone at 6 months to get their views on the usefulness of the supportive text messages. All patients will be similarly contacted regarding their satisfaction with their treatment using a semi-structured questionnaire. At 12 months, data related to each patient’s utilization of health services and their associated costs will be compiled from AHS administrative records by the blinded research associate.

### Primary Outcome Measure

1. Mean changes in the BDI scores from baseline for those with a primary diagnosis of Depression

2. CAD from baseline (since discharge from the Residential Addiction treatment program) for those with primary AUD.

### Secondary Outcome Measures for Patients With Primary AUD

1. Mean units of alcohol per drinking days for participants with AUD at 3, 6, 9 and 12 months, computed from self-reports.

2. Mean number of days to the first drink for participants with AUD, computed from self-reports.

3. Number of hazardous drinking days (drinking in excess of the safe limits) from baseline for participants with AUD at 3, 6, 9 and 12 months, computed from self-reports.

4. Mean changes in the scores on the OCDS from baseline for participants with AUD at 3, 6, 9 and 12 months.

5. Mean changes in the scores on the Alcohol Abstinence Self-Efficacy Scale from baseline for participants with AUD at 3, 6, 9 and 12 months

### Secondary Outcome Measures for Patients With Either Primary AUD or Primary Depression

1. Improved EQ-5D-5L scores from baseline. Health related Quality of Life will be measured in all groups at baseline, 3, 6, 9 and 12 months using the EQ-5D-5L (Canadian version).

2. Quantitative and qualitative data will be gathered from all participants in the intervention group using the semi-structured questionnaire in Multimedia Appendix 1 to assess their perceptions about how helpful or unhelpful the supportive text messages were to the course of their recovery.

### Other Exploratory Outcome Measures

1. Frequency of health services utilization at 3, 6, 9 and 12 months will be computed from self-reports and compared to the AHS administrative data on the frequency of health services utilization. We will be able to determine the usefulness and accuracy of self-report health utilization data in comparison with administrative data. Self-report data has the benefit of being available immediately.

2. The cost of health services utilization for patients in the intervention and control groups using the AHS administrative data.

3. Number of days absent from work at 3, 6, 9 and 12 months will be computed from self-reports.

### Statistical Methods

Since it is unlikely that this pilot study will be powered to detect sufficiently small clinically relevant differences, the primary purpose of the statistical analysis for this pilot study will be a thorough descriptive analysis of the data, with a view to understanding the amount and mechanism for any missing data. In particular one of the goals will be to determine the feasibility of long-term impact of supportive text message treatment and follow up in this population. An analysis of missing data may help to determine how to improve follow-up and retention of patients in the full study.

A secondary goal will be to examine the patterns of the longitudinal data, which will guide the planned analysis for a full study (e.g. using a Generalized Estimating Equation [GEE] approach or a generalized linear mixed models [GLMM] approach). In particular for each outcome measure we will look at the correlation between successive time points. Analyses will be done within each diagnostic group separately.

A final goal will be to produce summary statistics for the longitudinal data, which will provide estimates for future sample size calculations. A clinically meaningful difference has already been determined as in the stated hypotheses for the full study. From this pilot study we will be able to estimate the variability in the estimates that we would expect to see, which will help to guide the sample size estimation.

## Results

We expect the results for the primary, secondary and exploratory outcome measures to be available within 18 months of project commencement. It is expected that the results will shed further light on the feasibility of using automated supportive text message interventions in long-term care for patients with Depression and AUD. The results of the pilot trial will also inform the implementation of a full-scale multicenter clinical trial.

## Discussion

### Implementation Strategy

#### Overview

This research proposes a novel intervention that addresses a critical gap in care for Depression and AUD patients and their health care providers. The research seeks to test the delivery of responsive, effective, cost-efficient care that transcends geographic boundaries and makes available important psychological supports that would otherwise be inaccessible or significantly burdensome for patients to use, given their physical location or position on a waitlist. There has been excellent front-line support for this initiative during trial development. As part of an integrated knowledge translation approach [22], discussions between our team and health care practitioners (health addictions therapists, counselors, and nurses), directors, clinical leads, primary care practitioners, and others involved with the treatment of this patient population have generated important insights into the logistics and potential impact of delivering care through text messaging. The implementation plan for the research will occur in the phases described below.

#### Pre-Implementation Phase

The involvement of patients in the creation of therapeutic text messages alongside health care providers is a critical precursor for the intervention. Patient-direct interventions can enhance both the uptake of innovations into clinical practice [23, 24] and ensure that care is aligned with patient needs and values [25]. Ethics clearance has been received from the University of Alberta Ethics Review Board and AHS has granted operational approval for the project to proceed. Assembly and training of human resources to manage and support the study will also commence in this phase. The knowledge-to-action framework, based on planned action theory will guide the development and implementation of the research [26]. Staff working in both study centers will be recruited as research associates and trained to use assessment tools by the principal investigator who is a practicing psychiatrist.

The study will use internet-based text messaging infrastructure; our industry partner and AHS tech will be engaged at this stage to pilot test both the system and handsets to ensure full functionality and to troubleshoot potential issues in advance of the study commencement. Text message providers will be involved in testing and troubleshooting for potential issues. Advertisement of the research project targeted to patients will start, and communication about study commencement as well as tailored dissemination of study information to health care providers (general adult psychiatrists and primary care networks), zone leadership and operations will occur. Weekly, open research team meetings will commence during this phase to facilitate exchange, and to rapidly advance the work and/or troubleshoot as the need arises. The design of CBT and addictions-focused text messages will commence immediately between patients and practitioners. The intent of the text messages is to supplement therapist-patient interaction; therefore there will be a high degree of collaboration between patients and practitioners during this activity. Once the trial infrastructure is in place, a study commencement date will be determined and the research will be executed according to the details outlined in this protocol.

#### Implementation Phase

Implementation will commence with recruitment of patients who will undergo study orientation, baseline assessment, and data collection. The intervention will be randomized and delivered according to the methods outlined earlier. Scheduled, open research team meetings will provide a forum for practitioners who are supporting study participants. Given that the intervention is patient-focused, key measures arising from its use will be monitored as the study data is collected and assessed by the Trial Management Committee. Ongoing, scheduled communication between the recruitment sites and research team administrators will commence during this phase to support recruitment, address any challenges arising, and to optimize implementation. Feedback will be actively solicited from health care providers, zone leaders and operations through weekly open research team meetings and supplemented by brokering activity undertaken by research team staff. As the trial moves past the mid-study mark (and should trial data suggest it effective and feasible), discussions will commence concerning the scaling up of the intervention through a full-scale, multi-site randomized trial [27, 28, 29].

### Behavior Change

The paradigm for behavior change using text messages as a patient-direct intervention is consistent with a CBT approach and addictions counseling principles. These approaches will be rigorously evaluated to detect potential impact during the course of the research. We intend to have a less direct impact on practitioner behavior with the adoption of texting as we anticipate texts will be a desirable part of the suite of treatment and counseling tools available to support their work. There is a high degree of interest in this modality within the practitioner community; as such, the bulk of our interaction time with practitioners and counselors will be used to encourage bi-directional linkage and exchange between these clinicians and the research team in support of study participants. Given the automaticity of the messages, we do not anticipate the need for interventions to specifically modify practitioner behavior per se; we anticipate that our main role with respect to the practitioners will be to ensure they are well-supported with the research, appropriately apprised of all aspects of the technology and message delivery, consistent with a robust, integrated knowledge translation approach.

### Commitment

We are fortunate to have excellent commitment by administrative, clinical and operational-level leaders engaged and ready to implement the intervention. The possibility of increasing patient access and support, as well as addressing unmet patient health care needs during particularly critical periods is of great interest to the practitioner communities we have engaged thus far. There exist very strong relationships among practitioners, disciplines and programs in rural and remote areas of Alberta; we perceive a high level of team presence in meeting the needs of our patient group. We look forward to continuing to build meaningful, supportive relationships between our team and health care practitioners during the course of the trial, especially among primary care physicians and alcohol detoxification and treatment centers, in order to better serve the needs of this patient group. Our success in sustaining lasting impact is directly related to the relationships we engender among these important stakeholders. Given the automatic and less visual nature of the intervention, we have gathered together committed, dedicated research team staff to help foster strong relationship-building through deliberate linkage and exchange efforts.

### Training

We are fortunate to have assembled a team of research associates comprising CBT and addiction counselors and permanent staff from the study sites to execute this research in partnership with our research team. The primary intent of our team composition is to match the expertise requirements of the research and to facilitate knowledge translation through capacity and relationship building. The team was assembled to achieve an optimal mix of both content-specific and cross-content training expertise that could provide pragmatic experiences and mentorship opportunities for all involved. Our aim is to enable the kind of integrative skills development that will be required of future health services, addictions and mental health, knowledge translation, and health economics researchers. The study team will use several tools in addition to regular open research team meetings, including an access-restricted SharePoint platform (to enable intra-team interactions and to asynchronously support the sharing of study information and expertise), email interactions and the use of tele-/video-conferencing as the need arises. This study is deeply embedded within the Addiction and Mental Health Strategic Clinical Network (SCN) for Alberta, and thus has access to an extended provincial, national and international network of research expertise in the area, should any unforeseen challenges arise. The reach of the SCN network also provides an excellent opportunity for collaborators, staff involved with the study, and trainees to link and exchange with other experts with an interest in Addictions and Mental Health.

In developing the proposal, many important zone-level linkages were established with sites and practitioners in psychiatry and primary care. The research team and supports have made these relationships a key priority to enable the research and represent a deliberate effort to establish a strong foundation for bi-directional exchange that facilitates uptake and benefits patients. The opportunity to have hands-on experience with the implementation of patient-directed, technology-enabled change is a unique aspect of this study. As such, the team will encourage enduring capacity building by engaging clinicians, staff, operations and leadership through open invitations to attend and contribute to research meetings and training/dissemination events. As part of the end of project knowledge translation plan [30] and using the guidance of health care practitioners involved with the study, key messages will be developed from study findings for dissemination among all of the participating stakeholders. Finally, the research team will leverage the networking and dissemination opportunities offered through the SCN and those offered in partnership with provincial academic institutions in a way that is consistent with the nature and strength of the research findings. At the conclusion of the study, the team will host a knowledge exchange forum with patients, key stakeholders, decision makers, health care providers, and others, to share and encourage dialogue around the findings and, should the findings allow, as a way to explore opportunities for scaling up to a province-wide trial.

### Dissemination of Study Findings

We chose the highest quality design available to evaluate the intervention using a randomized control trial design. This level of evidence does not presume, but will certainly facilitate the potential to generalize study findings across Alberta. Evaluation using metrics at the patient, provider, and system (economic) level will be employed. We intend to enhance the impact of this research by targeting the dissemination of research findings at several levels (i.e. patient, practitioners, academics/researchers, as well as the health care systems, and health care economics communities). Patients involved with the research will be invited to help develop key messages arising from the work and help determine the format and modality for delivery. We will utilize existing communication channels in rural and remote areas to disseminate findings to health care practitioners on the advice of representative knowledge users on our research team. The support of the Addiction and Mental Health SCN and provincial Alcohol Networks and Detoxification Centers will be leveraged to share findings, given the broad community of stakeholders engaged through the network. Results will be shared at the annual SCN Connections meetings to encourage the consideration of technology to augment care.

We will disseminate findings to academics and researchers through peer-reviewed journals and provincial, national and international academic forums. Depending on the nature and strength of our findings, the trial is likely to be replicated in other centers because this population is ‘high needs’ and ‘hard to reach’, and accessibility, acceptability and effective care, in particular, are difficult care standards to attain.

Finally, given that we recognize the potential to transform care in a sustainable way, we believe the findings will ultimately inform and support administrative decision making with respect to scaling of the intervention within the province of Alberta and potentially beyond. To this end, we will plan a deliberate organizational engagement strategy to advance discussions about feasibility and effectiveness prior to the conclusion of the trial. This will help ensure the findings are a relevant part of decision-making processes in a way that is aligned with study findings as they emerge. We anticipate that this may help smooth the path for scaling at both leadership and operational levels, so that the potential impact of the intervention can reach patients in a timely fashion.

## Appendix

Multimedia Appendix 1 Patient survey: SMS text message AUD/depression relapse prevention study.

## Abbreviations

AASES: alcohol abstinence self-efficacy scale
AHS: Alberta health services
AUD: alcohol use disorder
BDI: Beck depression inventory
CAD: cumulative abstinence duration
CBT: cognitive behavior therapy
GEE: generalized estimating equation
GLMM: generalized linear mixed models
HRQOL: health-related quality of life
SCN: strategic clinical network
