# Supplementary material for: Supportive Text Messages to Reduce Mood Symptoms and Problem Drinking in Patients With Primary Depression or Alcohol Use Disorder: Protocol for an Implementation Research Study
Source: JMIR Res Protoc. 2015 May 15;4(2):e55. doi: 10.2196/resprot.4371 (PMC4814207; doi:10.2196/resprot.4371)
Supplement: Multimedia Appendix 1 [file resprot_v4i2e55_app1.pdf]

Appendix I
Patient Survey: SMS Text Message AUD/Depression Relapse Prevention Study

Patient ID________________ Date: ____________ Interviewer_____________________

1. When you received text messages from the treatment team how often did you read them?
Always ( ) Often ( ) Sometimes ( ) Rarely ( ) Never ( )

2. How did the text messages make you feel?
   - Annoyed with the team ( )
   - Supported by the team ( )
   - Indifferent ( )
   - Other (Please elaborate)…………………………………………………………………

3. How satisfied were you with the frequency of the text messages?
   Very satisfied ( )
   Satisfied ( )
   Not sure ( )
   Dissatisfied ( )
   Very Dissatisfied ( )

4. How often would you prefer to receive supportive text messages?
   Twice daily ( ) More frequently ( ) Less frequently ( ) None at all ( )

5. Did the text messages help you remain abstinent?
   Always ( ) Often ( ) Sometimes ( ) Rarely ( ) Never ( )

Please elaborate...
....
....

6. Did the text messages play any role in improving upon your mood?
   Yes ( ) No ( )

Please elaborate...
....
....

7. On a scale of 1 to 5 where 1 means ‘Not helpful’ and 5 means ‘Extremely Helpful’, how would you rate the areas that were addressed by the text messaging service? Please circle the appropriate number.

   a) Curbing cravings
      Not Helpful 1 2 3 4 5 Extremely Helpful

   b) Monitoring your mood
      Not Helpful 1 2 3 4 5 Extremely Helpful

   c) Coping with stress
      Not Helpful 1 2 3 4 5 Extremely Helpful

   d) Coping with loneliness
      Not Helpful 1 2 3 4 5 Extremely Helpful

   e) Avoiding gatherings that increase the desire to indulge in your addiction
      Not Helpful 1 2 3 4 5 Extremely Helpful

   f) Motivation for recovery from depression or alcohol abuse
      Not Helpful 1 2 3 4 5 Extremely Helpful

   g) Preventing a relapse of depression or alcohol abuse
      Not Helpful 1 2 3 4 5 Extremely Helpful

   h) Other (Please specify)
      Not Helpful 1 2 3 4 5 Extremely Helpful

8. If you had a friend who was undergoing treatment for problem drinking or depression would you recommend the SMS service?

   Most Certainly ( ) Probably ( ) Not Sure ( ) Certainly Not ( )

Please elaborate..
....

9. Overall, how satisfied are the experience of receiving daily text messages and weekly phone calls as part of your treatment.
   Very satisfied ( )
   Satisfied ( )
   Unsure ( )
   Dissatisfied ( )
   Very Dissatisfied ( )

Please elaborate..
....
....
